# Causal associations between liver enzymes and diabetic microvascular complications: A univariable and multivariable Mendelian randomization

**DOI:** 10.1371/journal.pone.0296894

**Published:** 2024-01-17

**Authors:** Yang Li, Qiu Zhang

**Affiliations:** Department of Endocrinology, First Affiliated Hospital of Anhui Medical University, Hefei, China; The First Hospital of Jilin University, CHINA

## Abstract

**Background:**

Observational studies show that liver enzymes are diabetes risk factors. However, previous observational investigations on the relationship between liver enzymes and diabetic microvascular complications produced contradictory results. The purpose of this research is to examine the independent causal effects of liver enzymes on diabetic microvascular complications.

**Methods:**

Univariable Mendelian randomization (UVMR) and multivariable Mendelian randomization (MVMR) were utilized to disentangle the causal effects. The genome-wide association study (GWAS) summary-level statistics were collected from the UK biobank and the FinnGen consortium. Single nucleotide polymorphisms (SNPs) were selected as genetic instruments with genome-wide significance (*p* < 5 ×10^−8^). Five UVMR approaches, including inverse variance weighted (IVW), Bayesian weighted Mendelian randomization, MR-Pleiotropy Residual Sum and Outlier (MR-PRESSO), weighted median, and MR-Egger, and three MVMR approaches, including the extended versions of IVW, MR-Egger, and the Q-minimization methods, were performed to evaluate the causal effects. The robustness of the MR results was further confirmed using several sensitivity analyses.

**Results:**

UVMR revealed that a genetically predisposed per standard deviation increase in serum alanine aminotransferase (ALT) level increased the risk of diabetic retinopathy (DR) in type 2 diabetes mellitus (T2DM) (IVW OR = 1.489, 95% CI = 1.206–1.772, *p* = 0.006). Likewise, serum aspartate aminotransferase (AST) levels showed similar results (IVW OR = 1.376, 95% CI = 1.115–1.638, *p* = 0.017). Furthermore, these effects were consistent after controlling for glycemia and blood pressure using MVMR analysis. Additionally, sensitivity analyses further strengthened the causality. However, no significant associations were found between alkaline phosphatase (ALP), gamma-glutamyl transferase (GGT), and diabetic microvascular complications.

**Conclusions:**

Robust evidence was demonstrated for an independent causal effect of serum ALT or AST concentration on the risk of DR in T2DM. Further investigations are necessary to elucidate the potential biological mechanisms and confirm their clinical significance for early prevention and intervention.

## 1 Introduction

Diabetes Mellitus (DM) has come to be recognized as a major health concern, demonstrating a continually rising occurrence worldwide. Type 2 DM (T2DM) is the predominant form of DM, constituting 90–95% of the total cases of DM [1]. Diabetic microvascular complications, including diabetic retinopathy (DR), diabetic nephropathy (DN), and diabetic peripheral neuropathy (DPN), can have a significant influence on a person’s quality of life and longevity [2]. DR is the primary contributor to visual impairment and blindness, particularly in developing countries, which is largely attributed to the growing occurrence of T2DM. Furthermore, DR is also associated with cardiovascular disease and cognitive impairment [3]. DN is the predominant factor contributing to end-stage kidney disease in the United States, representing between 30–50% of all reported cases. Additionally, it is estimated that nearly 30–40% of individuals with diabetes develop DN [4]. DPN can lead to an increased risk of foot ulceration and sexual dysfunction, thereby negatively impacting the overall quality of life [2]. However, the underlying biological mechanisms are not fully understood, nor are the risk factors. Therefore, it is essential to recognize the risk factors for diabetic microvascular complications for early prevention and intervention.

Liver enzymes, including alanine aminotransferase (ALT), aspartate aminotransferase (AST), alkaline phosphatase (ALP), and gamma-glutamyl transferase (GGT), have been demonstrated as risk factors for T2DM in previous research [5–7]. However, the previous observational studies between liver enzymes and diabetic microvascular complications remain unclear, including positive [8–10], negative [8, 10, 11], and non-existent [8, 10]. A recent Mendelian randomization (MR) analysis indicated a non-linear causal relationship of serum ALT level in renal and retinal complications in diabetes [12]. Nonetheless, it remains unclear whether the causal effects differ between T1DM and T2DM patients, and the associations between liver enzymes and risk factors for diabetic microvascular complications are not well understood. In recent years, the clinical significance of serum ALT concentration has been extended and is not limited to hepatocellular damage. For instance, an elevated ALT level measured in the circulation was found to be associated with several metabolic diseases [13–16], representing "liver metabolic function" [17]. An elevated concentration of ALT in the blood serum is also a noteworthy indicator of nonalcoholic fatty liver disease (NAFLD), a widely occurring chronic liver disorder that is highly prevalent in those suffering from T2DM [18]. Inspired by these findings, it is reasonable to disentangle the causal relationships between liver enzymes and diabetic microvascular complications.

MR is designed to obtain the causal effect of a specific risk factor on disease outcome using instrument variables (IVs) collected from genome-wide association studies (GWAS), which are strongly associated with the risk factor of interest and are randomly distributed at birth. Therefore, it could overcome the limitations of traditional clinical studies, such as unknown confounders, reverse causation, and measurement error [19]. However, the MR results may also be biased when genetic instruments show potential heterogeneity or pleiotropy. Univariable MR (UVMR) is a reliable approach to estimating the total causal effect of an exposure on the outcome when the selected instruments are not pleiotropic [20]. When multiple exposures have strong associations with each other and share some genetic instruments, multivariable MR (MVMR) can disentangle the direct effect of an exposure on the outcome from the confusion of other exposures [21]. It is essential to conduct sensitivity analyses in MR studies in order to estimate potential pleiotropy and reinforce the reliability of causal estimations.

The aims of this study are to investigate the independent causal effects of liver enzymes on diabetic microvascular complications using MR methods. We employed UVMR and MVMR approaches to determine the direct causal effects of liver enzymes (specifically ALT and AST) on T2DM with DR and also performed sensitivity analyses to ensure the robustness of the results.

## 2 Materials and methods

For the purpose of this research, de-identified participant study data that were made available to the public and ethical standards committee-approved were used. In this research, no additional ethical approval was needed. The Strengthening the Reporting of Observational Studies in Epidemiology using Mendelian Randomization (STROBE-MR) checklist was utilized to report MR studies clearly and transparently (S1 Appendix) [22].

### 2.1 Data source

**Fig 1:**
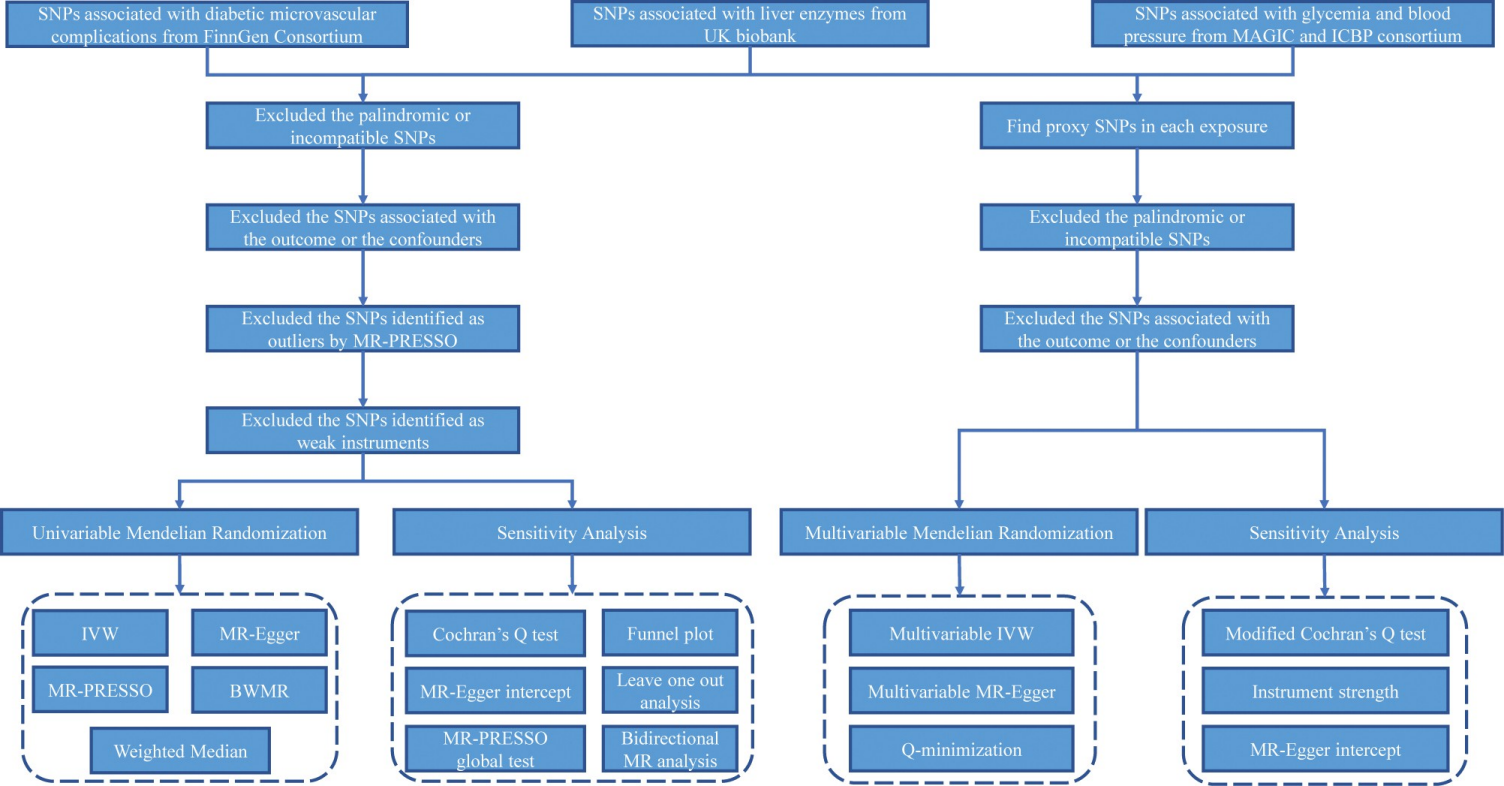
Study work flow of the UVMR and MVMR analyses revealed the causal associations between liver enzymes and diabetic microvascular complications.

An overview of the study design is presented in Fig 1. In this study, we enrolled four liver enzymes as exposures, including ALT, AST, ALP, and GGT. GWAS summary-level statistics of ALT, AST, ALP, and GGT, from the UK Biobank (https://www.ukbiobank.ac.uk/) [23, 24], were obtained from the Integrative Epidemiology Unit (IEU) OpenGWAS Project (https://gwas.mrcieu.ac.uk/) [25]. They were all of European ancestry and had an appropriate sample size, exceeding 430,000 individuals (S1 Table) [23]. All these liver enzymes were measured at the serum level using the unit per liter (U/L). The standard deviations (SD) of ALT, AST, ALP, and GGT in serum were 14.11, 10.65, 26.38, and 41.87, respectively.

For outcome summary data of diabetic microvascular complications, we focused on DR and DN due to their high incidence and disability rates in people with diabetes [3, 4]. Since different types of diabetes have different pathogenesis, we performed subgroup analyses to determine the causal effects between liver enzymes and diabetic microvascular complications in T1DM and T2DM. The summary-level statistics were all collected from the latest version of the FinnGen consortium (https://r9.finngen.fi/) [26], including DR (10,413 cases and 308,633 controls; ICD-10: H36.0), T1DM with DR (5,202 cases and 308,280 controls; ICD-10: E10.3), T2DM with DR (4,172 cases and 303,280 controls; ICD-10: E11.3), DN (4,111 cases and 308,539 controls; ICD-10: N08.3), T1DM with DN (1,579 cases and 303,280 controls; ICD-10: E10.2), and T2DM with DN (2,684 cases and 303,280 controls; ICD-10: E11.2).

Several risk factors for diabetic microvascular complications were taken into consideration to disentangle the direct causal effects when conducting the MVMR analysis. It is widely acknowledged that glycemia and blood pressure are the leading causes of the progression of DR and DN [3, 4]. The GWAS summary data for serum glycated hemoglobin, which could effectively represent the average blood glucose level over the previous three months, was retrieved from a study published recently [27]. For the blood pressure dataset, the summary-level genetic information of both systolic blood pressure (SBP) and diastolic blood pressure (DBP), from the International Consortium of Blood Pressure (ICBP), was extracted from the IEU database [28]. Details of the summary-level data are displayed in S1 Table.

### 2.2 Univariable Mendelian randomization

For UVMR analysis, it is necessary for IVs to fulfill the three assumptions below: (1) IVs are associated with the exposure; (2) IVs should be independent of the outcome; and (3) IVs must be independent of all confounders of the outcome [29]. A breach of these IV assumptions could result in biased causal estimations. To obtain effective IVs, we implemented a series of methods for selection. Firstly, we identified SNPs that were genome-wide significant (*p* < 5 ×10^−8^) and had linkage disequilibrium statistics (r^2^ < 0.001). Next, the SNPs, which were associated with the outcome or palindromic with intermediate allele frequencies, were excluded. The *F*-statistic of each SNP was then computed to evaluate whether the SNP was a weak instrument, set at a threshold of 10 [30]. Only the SNPs with an *F*-statistic over 10 were enrolled for further analysis. Furthermore, we also excluded the SNPs associated with confounding the outcome by using the PhenoScanner (http://www.phenoscanner.medschl.cam.ac.uk/) and the GWAS Catalog databases (https://www.ebi.ac.uk/gwas/) [31, 32]. In this study, we focused on DR and DN. Therefore, some critical confounders were taken into consideration, including hyperglycemia, hypertension, body mass index, and obesity. Finally, MR-Pleiotropy Residual Sum and Outlier methods (MR-PRESSO) were utilized to detect and remove outliers in the SNPs [33]. In addition, the SNPs lost in the outcome GWAS data were discarded.

To assess the total causal effects between exposures and outcomes, we applied several UVMR analytical methods, such as inverse variance weighted (IVW), Bayesian weighted Mendelian randomization (BWMR), MR-PRESSO, weighted median, and MR-Egger. The IVW model, which postulates that the IVs can only influence the outcome through a specific exposure, can yield a reliable result with balanced horizontal pleiotropy [34]. A random effects model of IVW was performed when substantial heterogeneity was detected [35]. Even in the presence of unbalanced pleiotropy or significant heterogeneity, the MR-Egger regression can produce a reliable estimation [36]. The weighted median results are trustworthy despite the fact that up to 50% of the IVs are invalid [37]. MR-PRESSO was employed to evaluate genetic pleiotropy and remove outliers to balance the horizontal pleiotropy and heterogeneity [33]. BWMR is able to accommodate the estimation of weaker effects and weaker horizontal pleiotropic effects while also exhibiting the ability to detect outliers resulting from the presence of a few significant horizontal pleiotropic effects [38].

### 2.3 Multivariable Mendelian randomization

When possible risk factors are taken into account, MVMR analysis, an extension of UVMR, can assess the direct causal effect between exposure and outcome [29]. The criteria for IVs enrollment are that the IVs should be associated with one of the exposures and not associated with either the potential confounders or the outcome [29]. The thresholds of *p*-value and r^2^ were the same as previously mentioned. Next, the proxies were found if the SNPs were absent in other exposures using the "TwoSampleMR" R package [39]. Furthermore, the palindromic or ambiguous SNPs were discarded. Finally, the SNPs associated with either the outcome or the confounders were also removed to balance the potential horizontal pleiotropy. Considering the possible relationship between liver enzymes, hyperglycemia, and high blood pressure, the extension frameworks of IVW and MR-Egger were utilized to undertake an MVMR analysis based on the non-overlapping samples of each exposure [21, 40]. Additionally, the Q-statistic minimization method, which provides robust causal effect estimation even when instruments are weak or exhibit pleiotropy, was implemented to make the results more reliable [41].

### 2.4 Mediation analysis

To explore the mediating effect between exposure, mediator, and outcome, a network (or two-step) MR analysis was conducted [42]. Network MR utilizes UVMR analyses to evaluate the effects of exposure on the outcome, exposure on the mediator, and mediator on the outcome, respectively. Subsequently, the mediating effect of exposure on the outcome via a specific mediator can be calculated using a public website tool (https://amplab.shinyapps.io/MEDCI/) based on the product of coefficients methods [43]. Finally, the mediation proportion was also computed. In the mediation analysis of this study, the IVW estimations from the UVMR analysis were used as the main results. All the procedures of UVMR analysis were similar, as previously mentioned.

### 2.5 Sensitivity analysis

In the UVMR analysis, several approaches were employed to further confirm the robustness of the findings. To detect the heterogeneity between single genetic instruments, we conducted Cochran’s Q test in the MR-Egger and IVW models [44]. A random effect IVW model was implemented when detecting substantial heterogeneity (*p*-value of Cochran’s Q < 0.05). MR-Egger regression was applied to assess the global pleiotropy [36]. Significant pleiotropy was detected when the *p*-value of the MR-Egger intercept was lower than 0.05. The MR-PRESSO global test could also detect pleiotropy when the *p*-value was lower than 0.05 [33]. To avoid reverse causation, bidirectional MR was employed. The implementation of the bidirectional MR followed the same procedures as the previously mentioned in UVMR [45]. Scatter plots could visualize the relationship between the effect of a single SNP on exposure and outcome. Funnel plots showed the distribution of SNPs and could display outlier genetic instruments. Leave-one-out analysis can be utilized to assess the extent to which a single SNP significantly contributes to the causal estimation, as determined by repeating the IVW analysis when individual SNPs are removed consecutively. The statistical power was ascertained through a publicly available web-based instrument (https://shiny.cnsgenomics.com/mRnd/) [46].

For MVMR analysis, the instrument strength and validity were computed utilizing a modified version of Cochran’s Q statistic. The two-sample conditional *F*-statistic of less than 10 indicated a weak instrument. Furthermore, we could not reject the null hypothesis of no heterogeneity and pleiotropy when the *p*-value of instrument validity was over 0.05 [47]. The extended version of MR-Egger was performed to determine the possible pleiotropy, with the intercept *p*-value being less than 0.05, indicating considerable horizontal pleiotropy [40].

### 2.6 Statistical analysis

The analyses were conducted using R software, version 4.1.2. All MR analyses were implemented based on the R packages "TwoSampleMR" (0.5.6), "MendelianRandomization" (0.6.0), "BWMR" (0.1.1), and "MVMR" (0.3.0) [38, 39, 41, 48]. A Bonferroni-adjusted *p*-value of less than 0.0021 (0.05/24) was deemed to be a statistically significant indicator of a causal association when multiple tests were conducted in our MR analysis. In contrast, a *p*-value between 0.0021 and 0.05 suggests a possible causal association. In addition, all the analyses were two-sided.

## 3. Results

### 3.1 Genetic instruments

In total, 600 SNPs, robustly and independently associated with the four liver enzymes, were selected as IVs, including 125 SNPs for ALT, 87 SNPs for AST, 252 SNPs for ALP, and 136 SNPs for GGT. Detailed information for the selected IVs of each exposure was presented in S2–S5 Tables. The *F*-statistics ranged from 29 to 24525 for individual SNPs, with mean values of 1816, 1964, 2250, and 2111 for ALT, AST, ALP, and GGT, respectively, indicating the strong strength of our selected genetic instruments. In the MVMR analysis, the two-sample conditional *F*-statistics were calculated and were all over 10 for ALT after conditioning on other exposures. However, the *F*-statistics were less than 10 for AST when controlling for DBP or glycated hemoglobin, indicating possible weak instrument bias to estimate causal effects in MVMR. For variability explanation, these genetic instruments could account for 17.95% variance of ALT, 20.85% of AST, 9.98% of ALP, and 2.24% of GGT by using GWAS summary level statistics [49]. As estimated by MR-Egger regression analysis, the *I*^*2*^_*GX*_ for the four liver enzymes were all over 0.98, suggesting a rare chance of violating the negligible measurement error (NOME) assumption. To perform the two-sample MR and reduce the potential bias, we conducted a series of selections of genetic instruments. Firstly, we excluded the SNPs that could not be found in the outcome dataset and those that were incompatible or palindromic (S6 and S7 Tables). Next, the SNPs that were significantly associated with the outcome or the cofounders were also discarded (S8 and S9 Tables). Finally, the SNPs detected as outliers by MR-PRESSO were removed to balance the horizontal pleiotropy (S10 Table). MVMR analysis requires that SNPs be at least associated with one exposure in the model. The subsequent selection process of SNPs was similar to that used in UVMR analysis. The information on genetic instruments for MVMR analysis can be found in S11 Table.

### 3.2 Univariable Mendelian randomization

**Fig 2:**
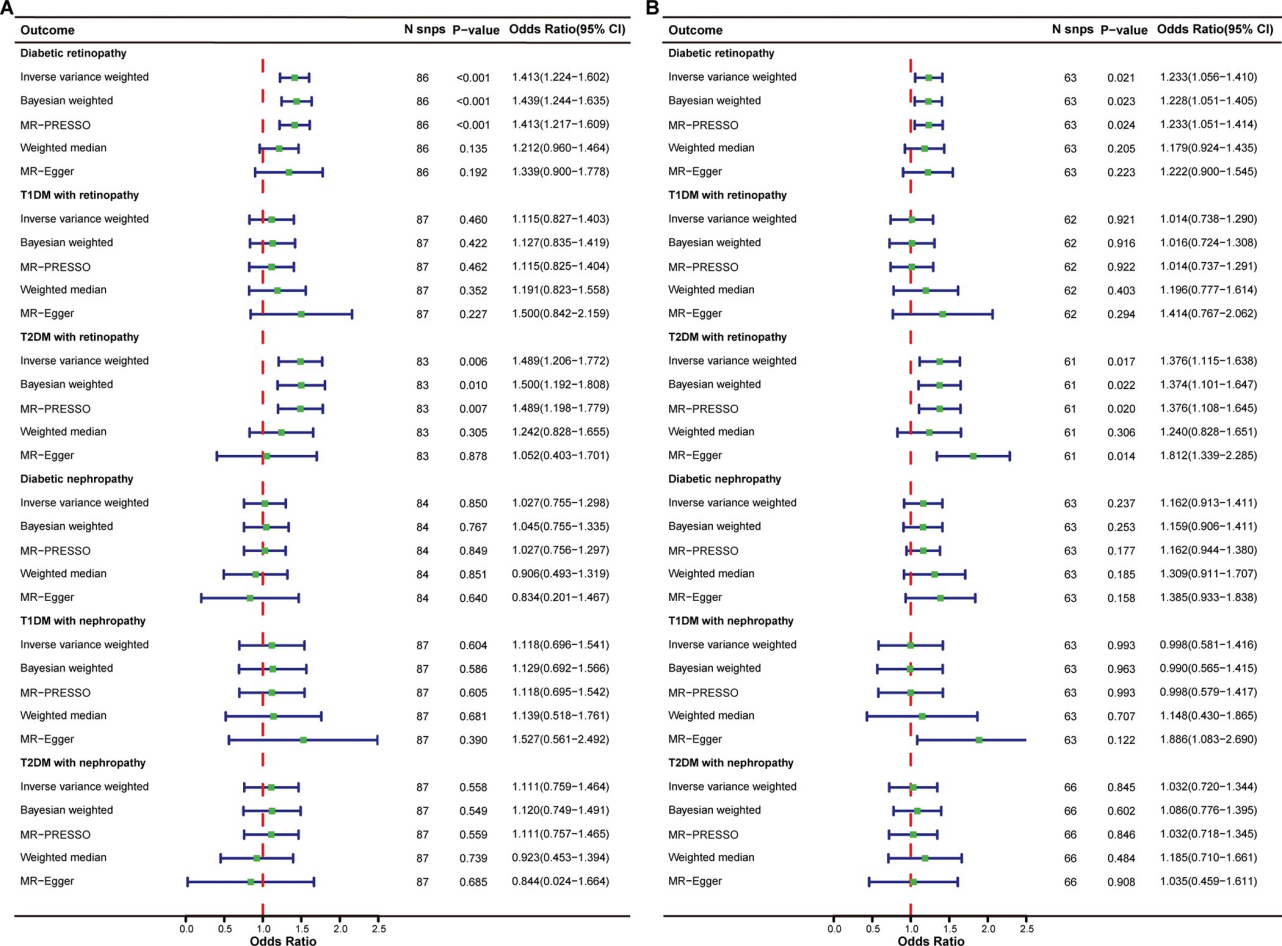
Summary of UVMR causal effects for serum ALT and AST levels on the risk of DR and DN. (A) Forest plot showed the UVMR estimations of serum ALT levels on the risk of DR and DN. (B) Forest plot showed the UVMR estimations of serum AST levels on the risk of DR and DN.

Using SNPs robustly and independently associated with ALT, UVMR revealed suggestive evidence that ALT increased the risk of DR overall (IVW OR = 1.413, 95% CI = 1.224–1.602, *p* < 0.001, per SD increase of ALT), but provided limited evidence for DN overall (IVW OR = 1.027, 95% CI = 0.755–1.298, *p* = 0.850) (Fig 2A). For subgroup UVMR analyses, we found a similar risk effect on T2DM with DR (IVW OR = 1.489, 95% CI = 1.206–1.772, *p* = 0.006) (Fig 2A). However, the causal effects of ALT on T1DM with DR, T1DM with DN, and T2DM with DN were not statistically significant (Fig 2A). When estimating the causal effects of ALT on DR overall and T2DM with DR, all five MR methods (IVW, BWMR, MR-PRESSO, Weighted Median, and MR-Egger) provided the same direction of causal estimations. Nevertheless, the weighted median and MR-Egger results demonstrated wider confidence intervals (Fig 2A).

From the UVMR analyses, we observed that increased AST positively affected the occurrence of DR overall (IVW OR = 1.233, 95% CI = 1.056–1.410, *p* = 0.021, per SD increase of AST) (Fig 2B). This effect was also significant in T2DM with DR (IVW OR = 1.376, 95% CI = 1.115–1.638, *p* = 0.017), but not in T1DM with DR (IVW OR = 1.014, 95% CI = 0.738–1.290, *p* = 0.921) (Fig 2B). The direction of causal effects was consistent across all five UVMR methods, further confirming the positive causal association between the AST and DR. The IVW results for the causal relationship between AST and DN overall showed limited evidence for the estimation (IVW OR = 1.162, 95% CI = 0.913–1.411, *p* = 0.237) (Fig 2B). No significant association was identified between either T1DM with DN (IVW OR = 0.998, 95% CI = 0.581–1.416, *p* = 0.993) or T2DM with DN (IVW OR = 1.032, 95% CI = 0.720–1.344, *p* = 0.845) and the elevation of AST (Fig 2B).

The UVMR results of ALP on DR and DN suggested a null causal effect. In detail, we found a slightly negative causal effect between ALP and DR overall (IVW OR = 0.965, 95% CI = 0.867–1.064, *p* = 0.481, per SD increase of ALP) and ALP and DN overall (IVW OR = 0.989, 95% CI = 0.852–1.125, *p* = 0.871) (S1A Fig). The causal estimations for GGT on DR overall (IVW OR = 1.076, 95% CI = 0.941–1.210, *p* = 0.289, per SD increase of GGT) and DN overall (IVW OR = 0.869, 95% CI = 0.654–1.084, *p* = 0.201) also suggested a null causal effect (S1B Fig). The subgroup analyses of ALP and GGT showed no significant results. Considering the inconsistent direction of causal effects and unsatisfactory *p*-values, we were unsure whether there was an actual causal relationship.

### 3.3 Multivariable Mendelian randomization

**Fig 3:**
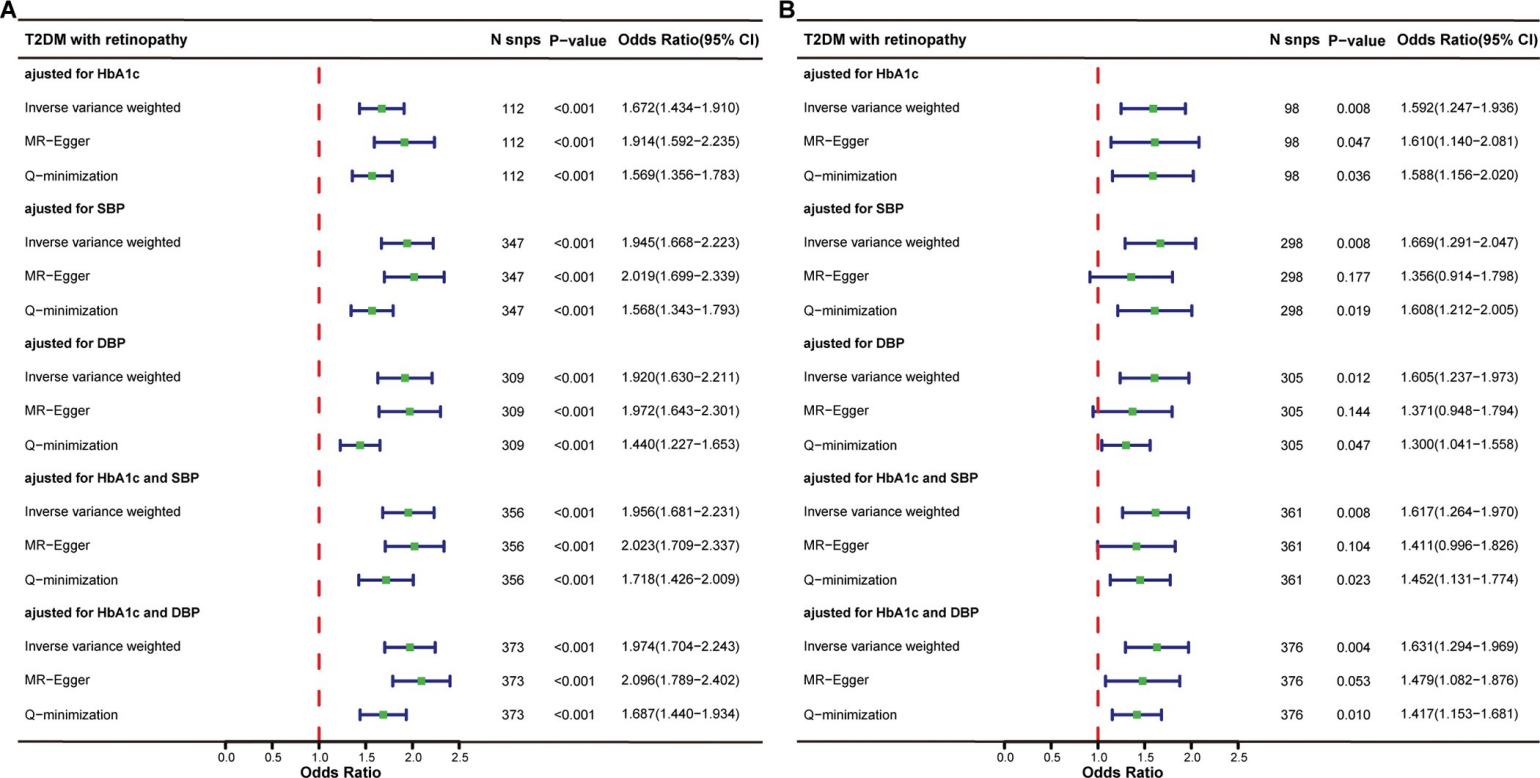
Summary of MVMR causal effects for serum ALT and AST levels on the risk of DR in T2DM patients when controlling for risk factors. (A) Forest plot showed the direct causal effect of serum ALT levels on the risk of DR in T2DM patients. (B) Forest plot showed the direct causal effect of serum AST levels on the risk of DR in T2DM patients.

In the MVMR analysis controlling for glycated hemoglobin, robust evidence was demonstrated for a direct causal effect of ALT (IVW OR = 1.672, 95% CI = 1.434–1.910, *p* < 0.001, per SD increase of ALT) and AST (IVW OR = 1.592, 95% CI = 1.247–1.936, *p* = 0.008, per SD increase of AST) on the risk of T2DM with DR (Fig 3A and 3B). In the MVMR analysis controlling for SBP, robust evidence was also demonstrated for a direct causal effect of ALT (IVW OR = 1.945, 95% CI = 1.668–2.223, *p* < 0.001) and AST (IVW OR = 1.669, 95% CI = 1.291–2.047, *p* = 0.008) on the risk of T2DM with DR (Fig 3A and 3B). In the MVMR analysis controlling for DBP, robust evidence was also demonstrated for a direct causal effect of ALT (IVW OR = 1.920, 95% CI = 1.630–2.211, *p* < 0.001) and AST (IVW OR = 1.605, 95% CI = 1.237–1.973, *p* = 0.012) on the risk of T2DM with DR (Fig 3A and 3B). After adjustment of both glycated hemoglobin and SBP, we observed positive direct causal effects of ALT (IVW OR = 1.956, 95% CI = 1.681–2.231, *p* < 0.001) and AST (IVW OR = 1.617, 95% CI = 1.264–1.970, *p* = 0.008) on T2DM with DR (Fig 3A and 3B). After adjustment of both glycated hemoglobin and DBP, we also observed positive direct causal effects of ALT (IVW OR = 1.974, 95% CI = 1.704–2.243, *p* < 0.001) and AST (IVW OR = 1.631, 95% CI = 1.294–1.969, *p* = 0.004) on T2DM with DR (Fig 3A and 3B). The direct causal estimations of multivariable MR-Egger regression were directionally consistent with the IVW results of ALT and AST when adjusting any of the confounders. In the causal relationship between ALT and T2DM with DR, the MR-Egger method provided strong evidence for the causal estimation, as all *p*-values were less than 0.001. However, it was not statistically significant when inferring the causal relationship between AST and T2DM with DR (Fig 3A and 3B).

### 3.4 Sensitivity analysis

In UVMR analyses, several heterogeneity and pleiotropy tests were implemented to determine whether the causal estimations violated the three essential assumptions of MR (Table 1). The Cochran’s Q test demonstrated limited evidence of heterogeneity for IVs in ALT and AST. The IVs, utilized to estimate the causal effect of ALT on T2DM with DR, showed limited heterogeneity in the IVW (*p* = 0.308) and MR-Egger model (*p* = 0.297). Similar results were found in AST on T2DM with DR in both the IVW (*p* = 0.372) and the MR-Egger models (*p* = 0.336). In addition, the scatter plots also displayed the low heterogeneity of the IVs (S2A–S2D Fig). For the directional pleiotropy tests, we did not observe significant pleiotropic effects through the MR-Egger intercept, as the *p*-values of the intercept were not significant in the causal relationships of ALT on T2DM with DR (*p* = 0.246) and AST on T2DM with DR (*p* = 0.159). After the outlier SNPs were excluded, the MR-PRESSO global test also indicated no significant pleiotropy in the causal estimations of ALT (*p* = 0.316) and AST (*p* = 0.275) on T2DM with DR. Additionally, the funnel plots showed symmetry, suggesting a balanced pleiotropy (S3A–S3D Fig). Leave-one-out results demonstrated that no individual SNP could significantly affect the causal estimation (S4A, S4B, S5A and S5B Figs). The statistical powers for the causal effects in the relationships of ALT and AST on T2DM with DR were satisfactory (power = 1.00). The bidirectional MR analysis suggested insufficient evidence to support a reverse causal association between serum ALT and AST levels and DR (S6A and S6B Fig). Taking all these results into consideration, we could conclude that the UVMR results were reliable and had limited bias.

**Table 1:** Sensitivity analysis for the univariable Mendelian randomization.

Exposure	Outcome	Heterogeneity *P* value		Pleiotropy *P* value		*F*-statistics	Power
		Inverse variance weighted	MR-Egger	MR-Egger intercept	MR-PRESSO global test		
Alanine aminotransferase	Diabetic retinopathy	0.066	0.075	0.789	0.081	127995	1.00
Alanine aminotransferase	Type 1 diabetes with retinopathy	0.002	0.002	0.324	<0.001	131135	0.73
Alanine aminotransferase	Type 2 diabetes with retinopathy	0.308	0.297	0.246	0.316	115813	1.00
Alanine aminotransferase	Diabetic nephropathy	0.503	0.518	0.477	0.514	124996	0.08
Alanine aminotransferase	Type 1 diabetes with nephropathy	0.401	0.416	0.481	0.419	129695	0.18
Alanine aminotransferase	Type 2 diabetes with nephropathy	0.143	0.151	0.466	0.164	130008	0.06
Aspartate aminotransferase	Diabetic retinopathy	0.085	0.099	0.949	0.100	100377	1.00
Aspartate aminotransferase	Type 1 diabetes with retinopathy	0.333	0.324	0.265	0.329	88172	0.07
Aspartate aminotransferase	Type 2 diabetes with retinopathy	0.372	0.336	0.159	0.275	93337	1.00
Aspartate aminotransferase	Diabetic nephropathy	0.928	0.928	0.356	0.931	99367	0.97
Aspartate aminotransferase	Type 1 diabetes with nephropathy	0.564	0.475	0.069	0.498	95396	0.05
Aspartate aminotransferase	Type 2 diabetes with nephropathy	0.244	0.272	0.990	0.264	108719	0.07

In MVMR analyses, several approaches were also employed to evaluate the heterogeneity and pleiotropy (Table 2). In the direct causal effect of ALT on T2DM with DR, the two-sample conditional *F*-statistics were all over 10, indicating the strong strength of selected instruments when conditioning on other exposures. The *p*-values for instrument validity were all over 0.05, demonstrating that we could not reject the null hypothesis of no heterogeneity and pleiotropy. The MR-Egger intercept also suggested a balanced pleiotropy. However, heterogeneity and pleiotropy were detected in the direct causal effect of AST on T2DM with DR. Weak instruments could make the IVW results biased when the two-sample conditional *F*-statistic was lower than 10. As a result, the Q-minimization method was performed to estimate the direct causal effect of AST on T2DM with DR when controlling for the confounders.

**Table 2:** Sensitivity analysis for the multivariable Mendelian randomization.

Exposure	Outcome	Adjustment	*F*-statistics	*P* value for instrument validity	*P* value for Egger intercept
Alanine aminotransferase	Type 2 diabetes with retinopathy	Glycated hemoglobin	62.12	0.129	0.223
Alanine aminotransferase	Type 2 diabetes with retinopathy	Systolic blood pressure	15.05	0.090	0.647
Alanine aminotransferase	Type 2 diabetes with retinopathy	Diastolic blood pressure	15.64	0.232	0.733
Alanine aminotransferase	Type 2 diabetes with retinopathy	Glycated hemoglobin and Systolic blood pressure	15.38	0.189	0.110
Alanine aminotransferase	Type 2 diabetes with retinopathy	Glycated hemoglobin and Diastolic blood pressure	14.61	0.099	0.421
Aspartate aminotransferase	Type 2 diabetes with retinopathy	Glycated hemoglobin	41.56	0.004	0.942
Aspartate aminotransferase	Type 2 diabetes with retinopathy	Systolic blood pressure	10.27	0.003	0.079
Aspartate aminotransferase	Type 2 diabetes with retinopathy	Diastolic blood pressure	9.77	0.015	0.141
Aspartate aminotransferase	Type 2 diabetes with retinopathy	Glycated hemoglobin and Systolic blood pressure	9.41	0.008	0.221
Aspartate aminotransferase	Type 2 diabetes with retinopathy	Glycated hemoglobin and Diastolic blood pressure	9.23	0.028	0.358

### 3.5 Mediation effect

Considering the high risk of glycated hemoglobin, SBP, and DBP on DR, mediation analysis was performed to explore whether serum ALT or AST concentration could increase the risk of T2DM with DR via these risk factors. Based on the network MR, the mediation effects were calculated and summarized in Table 3. We observed that all the mediation effects were not statistically significant due to a wide confidence interval. Furthermore, the proportion of mediation was extremely limited, ranging from 1.2% to 4.0%. Consequently, we could cautiously draw the conclusion that serum ALT or AST concentration may increase the risk of T2DM with DR partly via glycated hemoglobin, SBP, and DBP.

**Table 3:** Mediation analysis for ALT and AST on type 2 diabetes with retinopathy.

Exposure	Mediator	Outcome	Total effect^a^	Effect X^b^	Effect Y^c^	Mediation effect^d^	Mediation proportion
Alanine aminotransferase	Glycated hemoglobin	Type 2 diabetes with retinopathy	0.3980(0.2534,0.5426)	0.0142 (0.0020, 0.0264)	0.8602(0.5172,1.2031)	0.012 (-0.008, 0.04)	3.2% (0, 10.6%)
Alanine aminotransferase	Systolic blood pressure	Type 2 diabetes with retinopathy	0.3980(0.2534,0.5426)	0.7670 (0.4088, 1.1253)	0.0151(0.0101,0.0201)	0.012(0.001,0.027)	2.9% (0, 5.7%)
Alanine aminotransferase	Diastolic blood pressure	Type 2 diabetes with retinopathy	0.3980(0.2534,0.5426)	0.4605 (0.2494, 0.6716)	0.0103(0.0018,0.0188)	0.005(-0.003,0.016)	1.2% (0, 3.2%)
Aspartate aminotransferase	Glycated hemoglobin	Type 2 diabetes with retinopathy	0.3195(0.1860,0.4530)	-0.0261 (-0.0395, -0.0127)	0.8602(0.5172,1.2031)	NA	NA
Aspartate aminotransferase	Systolic blood pressure	Type 2 diabetes with retinopathy	0.3195(0.1860,0.4530)	0.8569 (0.3609, 1.3529)	0.0151(0.0101,0.0201)	0.013(-0.002,0.033)	4.0% (0, 8.5%)
Aspartate aminotransferase	Diastolic blood pressure	Type 2 diabetes with retinopathy	0.3195(0.1860,0.4530)	0.8582 (0.5807, 1.1356)	0.0103(0.0018,0.0188)	0.009(-0.005,0.027)	2.8% (0, 9.6%)

^a^Total effect: the effect of exposure on outcome.

^b^Effect X: the effect of exposure on mediator.

^c^Effect Y: the effect of mediator on outcome.

^d^Mediation effect: the effect of exposure on outcome via mediator.

## 4 Discussion

In this study, we integrated UVMR and MVMR approaches to determine the causal and independent effects of four liver enzymes on diabetic microvascular complications, including DR and DN. We observed that a genetically predisposed increase in ALT and AST was causally associated with the risk of T2DM with DR, indicating a potential risk factor. The causal estimates remained significant after adjusting for glycated hemoglobin, SBP, and DBP, highlighting an independent risk factor. However, similar causal effects were not observed for ALP and GGT on diabetic microvascular complications. Mediation analysis did not show significant mediating effects for ALT or AST on the risk of T2DM with DR via glycated hemoglobin, SBP, or DBP. Additionally, several sensitivity analyses were conducted and further confirmed the robust results when accounting for horizontal pleiotropy.

Some observational studies have reported the significance of ALT in diabetes and diabetic microvascular complications. A systematic review of 14 studies indicated that elevated levels of ALT in the blood was associated with an increased risk of T2DM (HR = 1.85, 95% CI = 1.57–2.18, *p* = 2.85 × 10^−13^, per log unit increase) [5]. Furthermore, a MR study confirmed the positive causal association between serum ALT levels and T2DM [50]. However, the relationship between ALT, DR, and DN remained unclear, including positive, negative, and non-existent correlations. In an observational study, D Song *et al*. reported that T2DM patients with NAFLD, who had an elevated serum ALT compared to T2DM patients without NAFLD, obtained a higher risk of DR in the subgroup analyses of Italy and India but a decreased risk for the Chinese, Korean, and Iranian populations, and suggested no relevance for the American population [8]. Furthermore, G Targher *et al*. revealed that NAFLD was associated with the prevalence of DN (OR = 2.4, 95% CI = 1.6–4.7, *p* < 0.001) [9]. In addition, M Afarideh *et al*. found a decreasing tendency of ALT in T2DM with DR (23.9 ± 10.9 U/L vs. 28.7 ± 23.0 U/L, *p* < 0.05) and T2DM with nephropathy (26.7 ± 14.1 U/L vs. 28.7 ± 23.0 U/L, *p* > 0.05) compared with T2DM patients free of microvascular complications [10]. By using the MR analysis, *Y Bi et al*. discovered a no linear causal association between ALT and DN or DR [12]. Yet, the sub-group analysis based on the classification of diabetes and MVMR analysis independent of confounding factors have not been extensively studied. The MR results of our study were in alignment with some of the previous reports. The UVMR results revealed a suggestive causal effect of ALT on T2DM with DR (OR = 1.489, 95% CI = 1.206–1.772, *p* = 0.006, per SD increase of ALT). The causal estimations remained significant and suggested a strong association (*p* < 0.001) after controlling for glycated hemoglobin, SBP, and DBP. The sensitivity analyses also demonstrated the robustness of the results. Taken together, we can conclude that genetically predisposed elevation of serum ALT concentration independently increases the risk of T2DM with DR and may be beneficial for early prevention and intervention.

Research was inadequate to substantiate a correlation between AST, T2DM, and microvascular complications of diabetes. The serum AST concentration was demonstrated as a weak risk factor for T2DM (RR = 1.13, 95% CI = 1.02–1.25, *p* = 0.021, per SD increase of AST) by a meta-analysis [6]. Furthermore, there was a decreasing trend of AST in T2DM with DR (21.5 ± 8.8 U/L vs. 23.9 ± 18.7 U/L, *p* > 0.05) and T2DM with nephropathy (21.5 ± 9.9 U/L vs. 23.9 ± 18.7 U/L, *p* > 0.05) compared to T2DM patients without either of them, despite the nonsignificant results [10]. Additionally, in a retrospective study of NAFLD and diabetic microvascular complications, W Lv *et al*. discovered that the occurrence of NAFLD was significantly lower in T2DM with diabetic microvascular complications, indicating possibly lower serum AST levels [11]. However, the UVMR and MVMR results showed a suggestive risk factor for AST on T2DM with DR (OR = 1.376, 95% CI = 1.115–1.638, *p* = 0.017, per SD increase of AST) independent of the glycated hemoglobin, SBP, and DBP. Although the sensitivity analyses presented robust results, we should cautiously conclude that the serum AST concentration is an independent risk factor for T2DM with DR and may be helpful for early prevention and intervention.

For the serum ALP and GGT, S Chen *et al*. reported in a prospective study that the elevation of serum ALP and GGT levels were significantly associated with the risk of T2DM when adjusting for multiple classical cofounders [7]. According to a recent investigation, M Afarideh *et al*. found that T2DM patients with either DR or DN had elevated serum ALP levels but limited evidence for a change in serum GGT levels [10]. In research on NAFLD and microvascular complications of diabetes, the incidence of NAFLD decreased in T2DM with microvascular complications, suggesting a possibly decreasing trend of serum ALP and GGT levels [11]. However, our findings showed that the serum ALP levels served as a protective factor in T2DM with DR or DN, and the elevated serum GGT levels increased the risk of T2DM with DR but decreased the risk of T2DM with DN. Nevertheless, there was limited evidence from which to infer the causal relationship, as all these results were not statistically significant.

The limitations of observational studies prevent researchers from exploring the causal associations between risk factors and diseases. Firstly, a critical shortcoming of all previous studies was that it was hard to avoid bias from the confounders of the risk factor. For instance, the patients with diabetes were different in terms of blood glucose management, duration of diabetes, blood pressure level, lifestyle, etc., which might contribute to the development of DR and DN [3, 4]. Furthermore, reverse causation is also a significant disadvantage of observational studies. Additionally, the sample sizes of the preceding studies were rather constricted, with the maximum sample size being 1,217 cases. Therefore, it might not provide sufficient statistical power to infer the causal relationship. Consequently, previous reports could not fully disentangle the causal associations of liver enzymes with DR and DN. Conversely, MR could surpass the restrictions that have been experienced in previous observational studies and more accurately elucidate the causal relationship between exposure and outcome.

Previous studies could provide some evidence for our hypothesis about the mechanisms between the ALT concentration in blood and the development of DR, although they still required further experimental investigation. Serum ALT concentration has long been acknowledged as a biomarker of liver injury. However, recent studies have demonstrated that serum ALT is a reliable predictor for T2DM [13], insulin resistance [14], coronary heart disease [15], and metabolic disease [16], indicating "liver metabolic function" [17]. Therefore, it is plausible to postulate that the ALT level measured in circulation could represent the relevant liver metabolic function beyond liver injury. Functionally, ALT played a significant role in liver alanine metabolism, which was the predominant liver glucogenic amino acid [51]. The hyperglycemic state in patients with T2DM is primarily attributed to changes in pancreatic function, peripheral glucose metabolism, and an overproduction of glucose by the liver [52]. Furthermore, a recent study demonstrated that the elevation of serum ALT levels was linked to hyperglycemia in T2DM patients and that silencing both ALT isoforms could retard hyperglycemia in T2DM mouse models via chronic glucocorticoid and glucagon signaling [53]. It has been found to be of great interest that hyperglycemia appears to be a key contributor in the development of retinal microvascular injury through multiple metabolic pathways, such as the increase of advanced glycation end-products, the hexosamine pathway, the protein kinase C pathway, and the polyol pathway [54]. Nevertheless, whether the elevated serum ALT level could increase the risk of DR in T2DM via hyperglycemia mechanically still needs further clinical and experimental validation in the future.

Understanding the limitations of our study could help us interpret the causal effects better. Firstly, the GWAS summary-level statistics utilized in this MR study are all of European ancestry. As a result, whether the causal relationship can be generalized to other ethnicities still needs further MR investigations of different ancestries. Secondly, the sample size of the DR and DN was limited (DR: 10,413 cases and 308,633 controls; DN: 5,202 cases and 308,280 controls), which may cause false-negative errors. Furthermore, the control group in the GWAS summary data for diabetic microvascular complications only consisted of non-diabetic participants, which could potentially influence the causal inference between liver enzymes and diabetic microvascular complications. Additionally, the subgroup MR analyses stratified by diabetes duration, sex, age, and other factors could not be conducted due to the absence of individual-level GWAS data. Finally, horizontal pleiotropy could not be completely avoided as the mechanisms of DR and DN are not fully explained, despite several sensitivity analyses demonstrating a null pleiotropy.

In conclusion, our study demonstrates that an increased serum ALT or AST level has a positive direct causal effect on the risk of DR in T2DM patients, independent of glycemia and blood pressure. Our findings could provide new evidence for early DR intervention in T2DM patients by monitoring and lowering serum ALT and AST concentrations. Further MR studies with sufficient sample size, large-scale genetic data, and more robust MR methods are needed to validate our results. We also expect that randomized controlled trials about reducing serum ALT and AST levels and the risk of DR in T2DM could be conducted to confirm their clinical significance in the future.

## Supporting information

S1 Fig Summary of UVMR casual effects for serum ALP and GGT levels on the risk of DR and DN. (A) Forest plot showed the UVMR estimations of serum ALP levels on the risk of DR and DN. (B) Forest plot showed the UVMR estimations of serum GGT levels on the risk of DR and DN.
(TIF)

S2 Fig Scatter plots displayed the casual effects of serum ALT and AST levels on the risk of DR overall and T2DM with DR. (A) is for ALT on DR overall. (B) is for ALT on T2DM with DR. (C) is for AST on DR overall. (D) is for AST on T2DM with DR.
(TIF)

S3 Fig The symmetry of funnel plots indicated a balanced pleiotropy. (A) is for ALT on DR overall. (B) is for ALT on T2DM with DR. (C) is for AST on DR overall. (D) is for AST on T2DM with DR.
(TIF)

S4 Fig Leave-one-out results demonstrated no individual SNPs that could significantly affect the causal effects of ALT on DR. (A) is for ALT on DR overall. (B) is for ALT on T2DM with DR.
(TIF)

S5 Fig Leave-one-out results demonstrated no individual SNPs that could significantly affect the causal effects of AST on DR. (A) is for AST on DR overall. (B) is for AST on T2DM with DR.
(TIF)

S6 Fig Bidirectional MR analysis revealed no reversal causal effects of DR on ALT and AST. (A) Forest plot showed the UVMR estimations of DR on ALT and AST. (B) Forest plot showed the UVMR estimations of T2DM with DR on ALT and AST.
(TIF)

S1 Table Detailed information for the datasets of the exposures and outcomes. (XLSX)

S2 Table Detailed information of genetic instruments for ALT. (XLSX)

S3 Table Detailed information of genetic instruments for AST. (XLSX)

S4 Table Detailed information of genetic instruments for ALP. (XLSX)

S5 Table Detailed information of genetic instruments for GGT. (XLSX)

S6 Table The SNPs that were lost in the GWAS summary data of the outcome. (XLSX)

S7 Table The SNPs that were palindromic or incompatible. (XLSX)

S8 Table The SNPs that were associated with the outcome. (XLSX)

S9 Table The SNPs that were associated with cofounders. (XLSX)

S10 Table The SNPs that were detected as outliers by MR-PRESSO. (XLSX)

S11 Table The SNPs that were utilized in MVMR. (XLSX)

S1 Appendix The STROBE-MR checklist of this MR study. (PDF)
