# Supplementary figures and images for: Causal associations between liver enzymes and diabetic microvascular complications: A univariable and multivariable Mendelian randomization
Source: PLoS One. 2024 Jan 17;19(1):e0296894. doi: 10.1371/journal.pone.0296894 (PMC10793938; doi:10.1371/journal.pone.0296894)

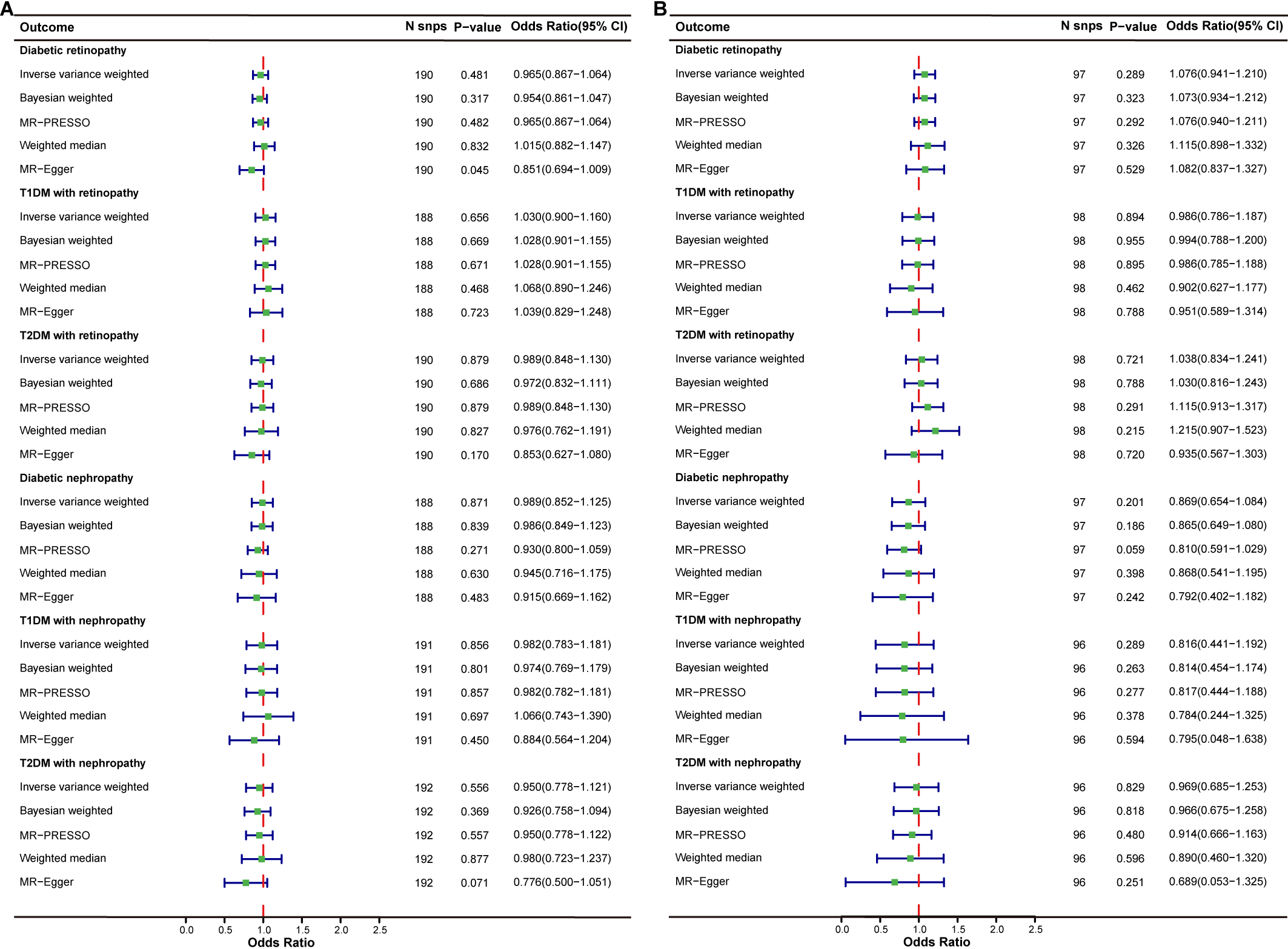

Supplement: S1 Fig — (A) Forest plot showed the UVMR estimations of serum ALP levels on the risk of DR and DN. (B) Forest plot showed the UVMR estimations of serum GGT levels on the risk of DR and DN. (TIF) [file pone.0296894.s001.tif]

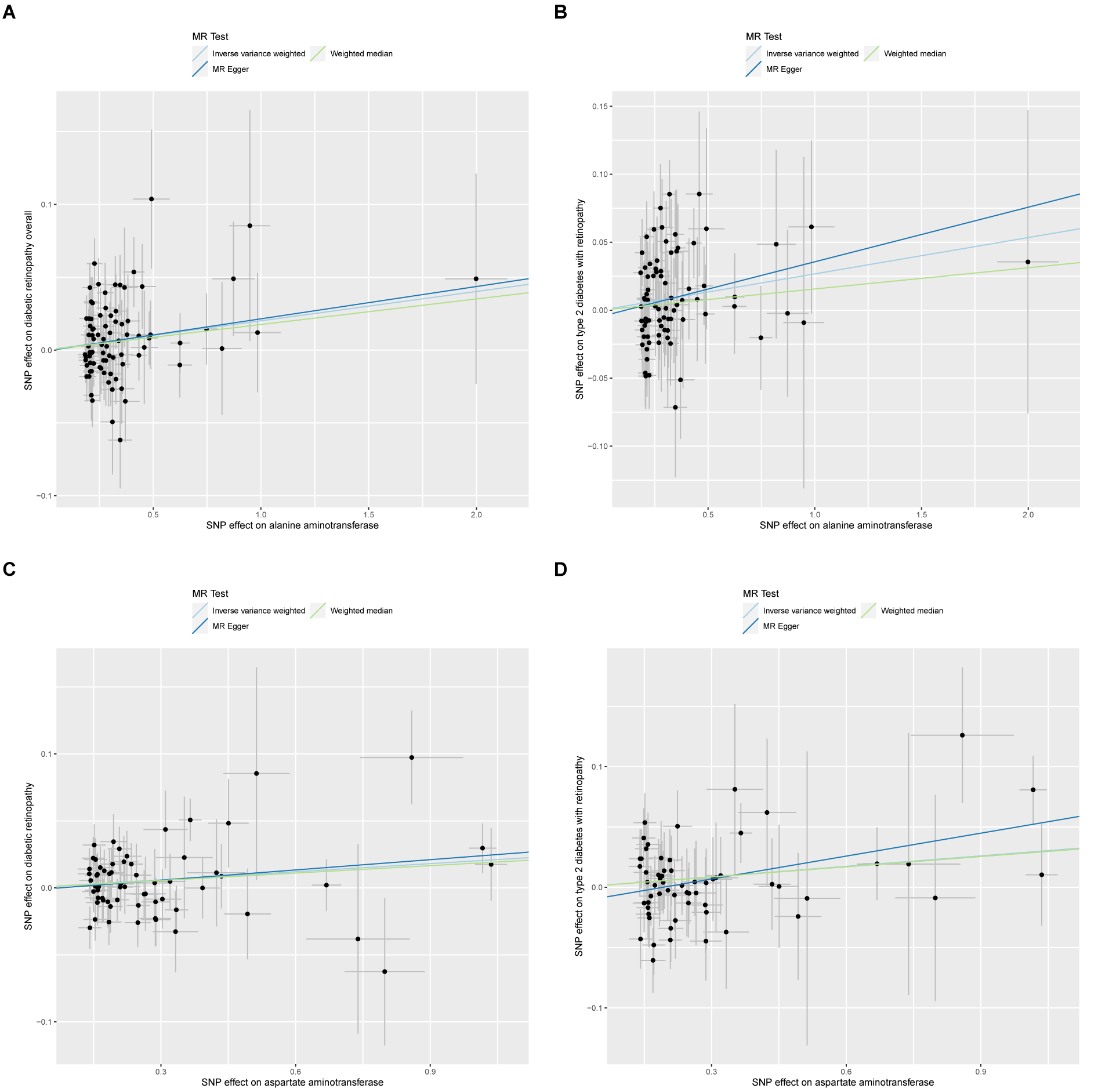

Supplement: S2 Fig — (A) is for ALT on DR overall. (B) is for ALT on T2DM with DR. (C) is for AST on DR overall. (D) is for AST on T2DM with DR. (TIF) [file pone.0296894.s002.tif]

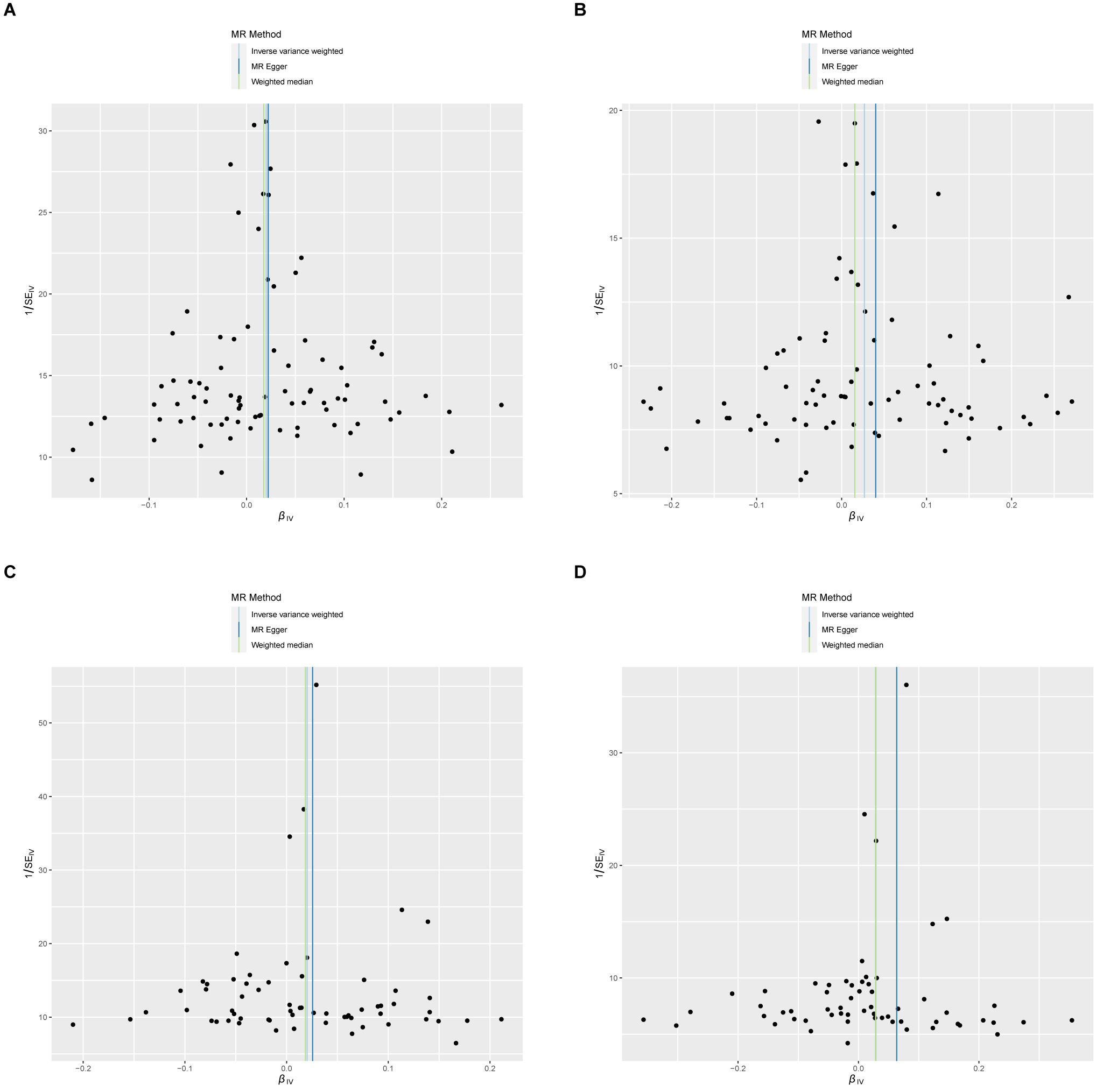

Supplement: S3 Fig — (A) is for ALT on DR overall. (B) is for ALT on T2DM with DR. (C) is for AST on DR overall. (D) is for AST on T2DM with DR. (TIF) [file pone.0296894.s003.tif]

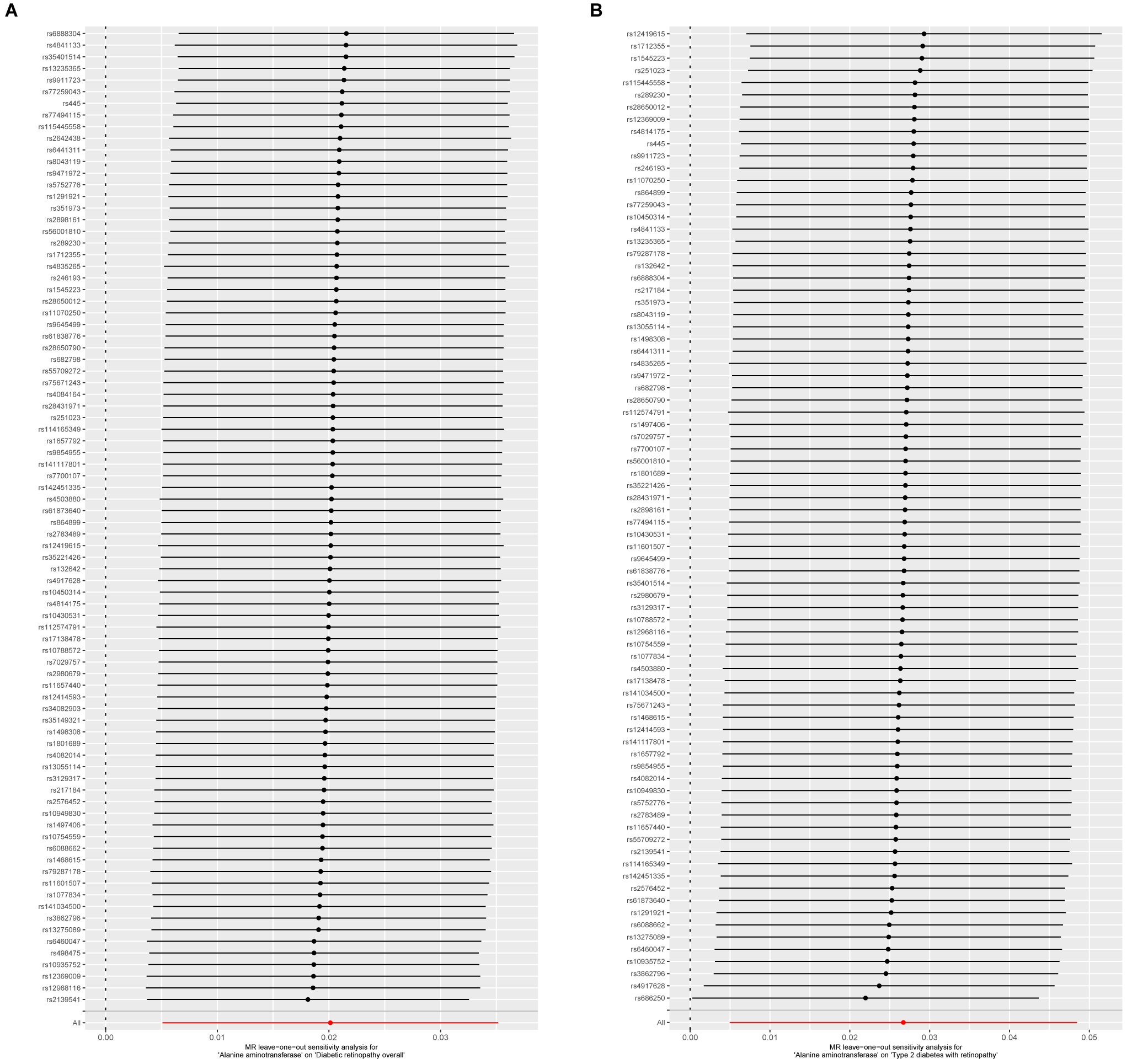

Supplement: S4 Fig — (A) is for ALT on DR overall. (B) is for ALT on T2DM with DR. (TIF) [file pone.0296894.s004.tif]

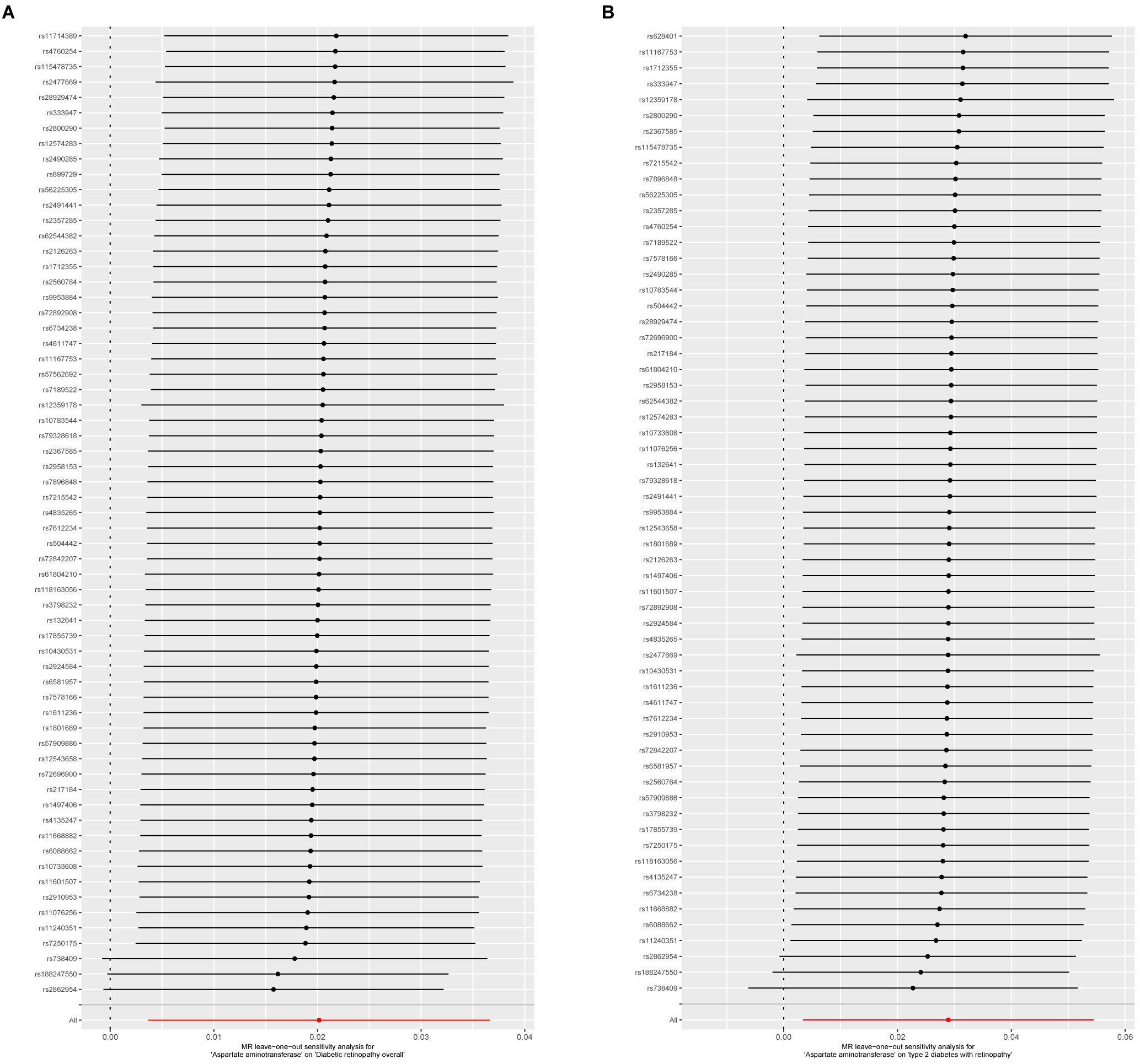

Supplement: S5 Fig — (A) is for AST on DR overall. (B) is for AST on T2DM with DR. (TIF) [file pone.0296894.s005.tif]

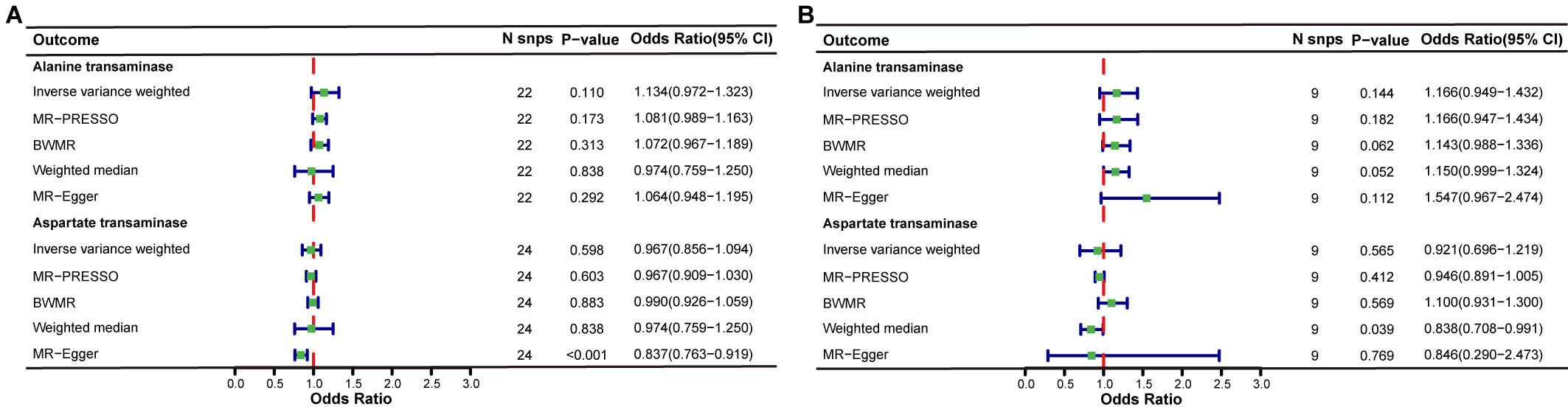

Supplement: S6 Fig — (A) Forest plot showed the UVMR estimations of DR on ALT and AST. (B) Forest plot showed the UVMR estimations of T2DM with DR on ALT and AST. (TIF) [file pone.0296894.s006.tif]
